# CRISPR-Cas for Fungal Genome Editing: A New Tool for the Management of Plant Diseases

**DOI:** 10.3389/fpls.2019.00135

**Published:** 2019-02-15

**Authors:** Isabel Vicente Muñoz, Sabrina Sarrocco, Luca Malfatti, Riccardo Baroncelli, Giovanni Vannacci

**Affiliations:** ^1^Department of Agriculture, Food and Environment, University of Pisa, Pisa, Italy; ^2^Spanish-Portuguese Center for Agricultural Research (CIALE), Department of Microbiology and Genetics, University of Salamanca, Villamayor, Spain

**Keywords:** genome editing, CRISPR-Cas9, filamentous fungi, biocontrol, plant diseases management, fungal pathogens, beneficial fungi

## Background

Fungal pathogens are the main factors responsible for the most severe diseases affecting plants, leading to significant reduction in yield and crop quality and causing enormous economic losses worldwide. It is estimated that around 30% of the emerging diseases are caused by fungi (Giraud et al., 2010) thus requiring new strategies to improve their management. Biological control approach, frequently referred to the use of non-pathogenic microbial antagonists or products derived from their metabolism, represents a valid and promising alternative under a more ecological perspective to reduce the activities and to control populations of target pathogens (Singh, 2016). However, although the use of antagonists belonging to species different from that of the pathogen has been successfully reported, the use of competitors belonging to the same species of the pathogen is not widespread. A biocontrol strategy based on competition for space and nutrients and/or the induction of plant defenses against virulent pathogens performed by attenuated or avirulent pathogens (Ghorbanpour et al., 2018) could, therefore, be considered a valid alternative.

## The Results So Far

Veloso et al. (2015) reported the use of an avirulent isolate of *Fusarium oxysporum* to reduce Verticillium wilt severity in pepper, through competition and induction of the plant defense responses. A similar approach was described by Salazar et al. (2012) for the management of anthracnose in strawberries. The avirulent isolate F7 of *Colletotrichum fragariae* conferred full protection from the infection caused by *C. acutatum* and also enhanced plant resistance against *Botrytis cinerea* through the induction of plant defense responses. Similarly, the use of an attenuated *Verticillium nigrescens* isolate reduced cotton wilt caused by a virulent isolate of *V. dahliae* (Vagelas and Leontopoulos, 2015).

(Aimé et al., 2013) used an avirulent isolate of *F. oxysporum* to combat *F. oxysporum* f. sp. *lycopersici* to reduce Fusarium wilt by priming a Salicylic-dependant signaling defense on tomato plants. The use of an avirulent strain of *Valsa mali* var. *mali* reduced the infection rate of apple tree canker caused by the virulent strain LXS080601 from 97 to 41% (Zhang et al., 2014) on apple callus. In 1993 as regards the mycotoxigenic fungi, Cotty and Bayman (1993) suggested the use of a non-aflatoxigenic isolate of *Aspergillus flavus* to control the development of aflatoxigenic strains in maize kernels by competitive exclusion and this strategy today is commercially applied in several countries (Ojiambo et al., 2018).

However, the selection of suitable isolates to be used as potential antagonists from the local fungal community often takes (long) time for identification and screening. Selection within a great number of isolates based on morphological, physiological and genetic features is usually required, followed by an *in vivo* screening against the pathogen on a real disease scenario. An interesting alternative to easily and quickly obtain new genotypes able to act as biocontrol agents, could be the induction of genetic mutations in the virulent genotypes, providing new avirulent strains that can compete directly with the virulent ones or induce plant defense responses (Ghorbanpour et al., 2018). The application of genetic transformation techniques to silencing genes putatively involved in pathogenicity has been widely used to uncoil the role of these genes in the establishment and development of the infection processes (Johnson et al., 2018). However, the disruption of a gene function usually involves the integration in the genome of foreign DNA sequences used as reporter genes in order to select transformants, leading to the generation of antibiotic-resistant or fluorescent strains. These genetic modifications represent a major constraint for their use in field.

## The Genome-Editing Era: State of the Art and Perspectives for the Management of Plant Diseases

The arrival of the CRISPR-Cas9 (Clustered Regularly Interspaced Short Palindromic Repeats – CRISPR Associated protein 9) genome-editing technique enabled researchers to modify genomic sequences in a more precise way (Knott and Doudna, 2018). CRISPR-Cas9 Type II system uses two principal components for gene targeting and cleavage: the RNA guide (sgRNA) and the Cas9 endonuclease. The sgRNA consists of a simple chimeric strand of RNA, which leads Cas9 up to the localization in the genome of the target gene, whose expression has to be blocked. Cas9 is able to bind the DNA and to produce a double strand break (DSB) in the target gene. The DSB then induces the activation of one of the DNA cellular reparation systems, the Non-Homologous End-Joining (NHEJ) system, that can re-join the ends of the DSB without introducing errors or, unlikely, giving rise to insertions or deletions of nucleotides during the repair. These InDels led to changes in the gene reading frame producing non-sense sequences or causing the appearance of premature stop codons, thus blocking the transcription of the target gene (Bono et al., 2015). In most cases the application of the technique in filamentous fungi consisted of a proof of concept of its feasibility (see Table 1). The system has the advantage that once implemented in the organism, it is possible to change the target gene by changing the sgRNA spacer sequence, as well as silencing several genes simultaneously by transforming the cell with different sgRNAs along with Cas9 (Hsu et al., 2015). Nevertheless, the most interesting advantage is that it allows to perform marker-free deletions by using transient expression plasmids that can self-replicate only under antibiotic pressure (Katayama et al., 2015, Nødvig et al., 2015; Schuster et al., 2016; Zhang et al., 2016; Liu et al., 2017; Wenderoth et al., 2017; Weyda et al., 2017; Wang et al., 2018). The use of CRISPR-Cas not only provides a time-saving path to perform genomic functional analyses, but also could provide new fungal genotypes, that can be used as potential competitors of plant pathogens and/or in the priming of plant defense responses.

**Table 1:** Application of CRISPR-Cas9 for gene-silencing in filamentous fungi.

**Fungal species**	**Edited gene**	**Aim**	**References**
*Alternaria alternata*	Polyketide synthase A (*pksA*) 1,3,8-THN reductase (*bmr2*)	Proof of concept	Wenderoth et al., 2017
*Aspergillus aculeatus* *Aspergillus brasilensis* *Aspergillus carbonarius* *Aspergillus luchuensis* *Aspergillus nidulans* *Aspergillus niger*	Polyketide synthase (albA) Laccase (yA)	Proof of concept	Nødvig et al., 2015
*Aspergillus carbonarius*	Pigment biosynthetic gene (*ayg1*)	Proof of concept	Weyda et al., 2017
*Aspergillus fumigatus*	Polyketide synthase P (*pksP*)	Proof of concept	Zhang et al., 2016
*Aspergillus nidulans* *Aspergillus niger* *Aspergillus oryzae*	Laccase (*yA*) Polyketide synthase (*albA*) Polyketide synthase (*wA*)	Proof of concept	Nødvig et al., 2018
*Aspergillus niger*	AMP-dependent synthetase and ligase α-aminoadipate-semialdehyde dehydrogenase Aldo/keto reductase Alcohol dehydrogenase, zinc-binding Short-chain dehydrogenase/reductase FAD-dependent oxidoreductase Mandelate racemase/muconate lactonizing enzyme d-isomer specific 2-hydroxyacid dehydrogenase	Production of galactaric acid	Kuivanen et al., 2016
*Aspergillus niger*	Polyketide synthase (*albA*)	Proof of concept	Zheng et al., 2018
*Aspergillus oryzae*	Polyketide synthase (*wA*) Conidial laccase (*yA*) Orotidine 5-phosphate decarboxylase (*pyrG*)	Proof of concept	Katayama et al., 2015
*Fusarium graminearum*	Histidine kinase 1 (*os1*) Trichodiene synthase (*tri5*)	Proof of concept	Gardiner and Kazan, 2018
*Fusarium oxysporum*	Orotate phosphoribosiltransferase (*ura3, ura5*) Polyketide synthase 4 (*pks4*)	Proof of concept	Wang et al., 2018
*Ganoderma lucidum* *Ganoderma lingzhi*	Orotate phosphoribosyltransferase (ura3)	Proof of concept	Qin et al., 2017
*Myceliopthora thermophila* *Myceliopthora heterotalica*	Carbon catabolite repression transcription factor (*cre-1*) Endoplasmic reticulum stress regulator (*res-1*) β-glucosidase (*gh1-1*) Alkaline protease (*alp-1*)	Enhancement of lignocellulase production	Liu et al., 2017
*Neurospora crassa*	Carbon catabolism repressor (*cre-1)*	Enhancement of cellulase production	Matsu-ura et al., 2018
*Penicillium chrysogenum*	Polyketide synthase (*pks17*)	Proof of concept	Pohl et al., 2016, 2018
*Pyricularia oryzae*	Scytalone dehydratase (*sdh*) suppressor of RAD six (*sdr2*)	Proof of concept	Arazoe et al., 2015
*Trichoderma reesei*	Transcription factor in cellulase biosynthesis (*clr2*) Orotate phosphoribosyltransferase (*ura5*) Gene putatively involved in glucose signaling and carbon catabolism repression (*vib1*) Methyltransferase (*lae1*)	Proof of concept	Liu et al., 2015
*Sclerotinia sclerotiorum*	Oxaloacetate acetylhydrolase (*Ssoah1*) Polyketide synthase (*Sspks13*)	Proof of concept Pathogenicity test	Li et al., 2018
*Ustilago maydis*	Central regulator of pathogenic development (*bW2*) (*bE1*)	Proof of concept	Schuster et al., 2016

One possible scenario for the application of CRISPR-Cas9 silenced mutants could be Fusarium Head Blight (FHB), one of the most destructive diseases of grain cereal crops worldwide caused by different *Fusarium* spp., with *F. graminearum* and *F. culmorum* as the most common and aggressive agents. In FHB, while yield loss derives from sterility of infected florets, grain quality reduction is mainly due to the accumulation of trichothecenes—coded by the fungal *tri* genes cluster—highly toxic for humans and animals. Previous studies reported that iRNA (interference RNA) Δ*tri6* mutants of *F. culmorum* showed reduced disease indices ranging from 40 to 80% on durum wheat (Scherm et al., 2011). In addition, classic knocked-out Δ*tri5* and Δ*tri6* mutants of *F. graminearum* were unable to spread the disease to the adjacent spikelets and grains on wheat and corn, respectively, and also induced plant defense responses (Ravensdale et al., 2014). Likewise, Δ*map1* mutants of *F. graminearum* showed two-fold reduction of mycotoxin production and were unable to produce perithecia as well as to penetrate in wheat tissues, while the ability to colonize the straw was not affected (Urban et al., 2003). Considering that competition for space and nutrients between virulent and non-virulent strains could reduce the disease, the field release of non-virulent CRISPR-mutant strains of *F. graminearum* and *F. culmorum* might help to control the incidence of FHB

Another contribution of CRISPR-Cas9 is the production of well-known antagonists with enhanced biocontrol aptitudes achieved through genome-editing (Vicente Muñoz et al., 2017). For example, species belonging to genus *Trichoderma* have been considered outstanding biocontrol agents able to reduce the disease severity (Sarrocco et al., 2013), not only by constraining the growth of the phytopathogens (Sarrocco et al., 2009), even killing them, but also by eliciting the plant defense responses (Fiorini et al., 2016; Sarrocco et al., 2017). One of the mechanisms through which these fungi can antagonize phytopathogenic fungi is the release of a wide arsenal of cell-wall degrading enzymes and secondary metabolites such as antibiotics, among others (Khalid, 2017).

Genetic engineering of the metabolic pathways that trigger the biosynthesis of secreted proteins and secondary compounds could provide new fungal strains with enhanced biocontrol activity. Previous studies reported that it is possible to achieve the same effect through the silencing of negative regulatory elements, signal-transduction components or genes belonging to contiguous metabolic networks, thus, redirecting metabolite flow and biosynthesis or supressing the feedback inhibition by which its production could be regulated (Bailey, 1991). For example, Δ*tvk1* mutants of *T. virens* displayed enhanced biocontrol activity against *R. solani*, in addition to an increased expression of mycoparasitism-related genes and overproduction of lytic enzymes (Mendoza-Mendoza et al., 2003). Likewise, four knockout mutants in SSCPs (small secreted cysteine rich proteins)-encoding genes of *T. virens* showed greater ability to induce ISR (Induced Systemic Resistance) on corn against *Cochliobolus heterostrophus* than the wild type (Lamdan et al., 2015). Another example of biocontrol enhanced ability was described by Reithner et al. (2005) in *T. atroviride*, in which the Δ*tga1* mutants exhibited an overproduction of antifungal secondary metabolites. Similarly, the Δ*tmk1* mutants of *T. atroviride* showed overproduction of 6-pentyl-pyrone and peptaibols, resulting in an enhanced antifungal activity and increased protection of bean plants against *Rhizoctonia solani* (Reithner et al., 2007). On the other hand, biosynthesis of secondary metabolites is often carried out by clustered genes whose expression could be induced by environmental conditions. However, in many cases these clusters are silent and their activation cannot be achieved (Osbourn, 2010). Bok et al. (2009) demonstrated that the silencing of a transcription factor involved in the methylation of lysine 4 of the histone H3 in *Aspergillus nidulans* activated the expression of cryptic clusters and yielded novel secondary metabolites. The silencing of *ace1* gene induces the up-regulation of four polyketide biosynthetic gene clusters in *T. atroviride*, leading to an increase in the production of antibiotics and other secondary metabolites that clearly enhanced its potential as biocontrol agent against *F. oxysporum* and *R. solani* (Fang and Chen, 2018). Following this approach, it is possible to induce the activation of unknown clusters in beneficial fungi by using CRISPR-Cas9, allowing the discovery of new secondary metabolites that could interact with plants or phytopathogens. This could result in new interesting biocontrol strains to be released in field avoiding the introduction of transgenes in the environment.

## Conclusions

The availability of novel or the improvement of known techniques that are safer for people and the environment is of outmost importance to guarantee food safety and security especially in those countries where famine is still an important issue (Vurro et al., 2010). A novel technique that allows the production of precise knock-out mutants without the insertion of foreign DNA in a saprotrophic/pathogenic fungus opens new possibilities of controlling plant pathogens. The use of such edited fungal strains needs a correct strategy to minimize possible risks. The major risk related to the release of a mutant strain is the rise, in the field, of novel combinations of pathogenesis/fitness related genes following the sexual or parasexual cycle. The genetic background of an edited isolate and its wild type is exactly the same, except for the edited gene, thus novel combinations of genes are not conceivable. Anyway, to further reduce such possibility, we can imagine a strategy of deployment that includes 1) the gene edit of the most prevalent genotype of the pathogen/saprotroph in the release area; 2) the editing of more than one gene in the same metabolic pathway and 3) the editing also of the idiomorphs and /or the HET genes, to make sexual or parasexual recombination (including the re-gain of virulence) even less likely.

Anyway, the application of novel techniques and the release of new products need, as usual, to be evaluated for their safety and to be accepted by populations. A recent sentence of the Court of Justice of the EU stated that edited organisms, even if they do not contain alien DNA, have to be subjected to the rules set up for Genetically Modified Organisms. This is not the place to discuss this issue, but it is high time, in EU at least, to reconsider the whole GMO legislation.

## Author Contributions

All authors listed have made a substantial, direct and intellectual contribution to the work, and approved it for publication.

### Conflict of Interest Statement

The authors declare that the research was conducted in the absence of any commercial or financial relationships that could be construed as a potential conflict of interest.
